# Fabrication of Carbohydrate Chips Based on Polydopamine for Real-Time Determination of Carbohydrate–Lectin Interactions by QCM Biosensor

**DOI:** 10.3390/polym10111275

**Published:** 2018-11-16

**Authors:** Kun Shang, Siyu Song, Yaping Cheng, Lili Guo, Yuxin Pei, Xiaomeng Lv, Teodor Aastrup, Zhichao Pei

**Affiliations:** 1 Shaanxi Key Laboratory of Natural Products & Chemical Biology, College of Chemistry & Pharmacy, Northwest A&F University, Yangling 712100, China; xinongkeda@nwafu.edu.cn (K.S.); ssyu137@163.com (S.S.); 2014014895@nwsuaf.edu.cn (Y.C.); 18337149831@163.com (L.G.); lxmhsd@163.com (X.L.); 2 Attana AB, SE-11419 Stockholm, Sweden; teodor.aastrup@attana.com

**Keywords:** polydopamine, amino-carbohydrates, carbohydrate chips, QCM biosensor, carbohydrate–lectin interactions

## Abstract

A novel approach for preparing carbohydrate chips based on polydopamine (PDA) surface to study carbohydrate–lectin interactions by quartz crystal microbalance (QCM) biosensor instrument has been developed. The amino-carbohydrates were immobilized on PDA-coated quartz crystals via Schiff base reaction and/or Michael addition reaction. The resulting carbohydrate-chips were applied to QCM biosensor instrument with flow-through system for real-time detection of lectin–carbohydrate interactions. A series of plant lectins, including wheat germ agglutinin (WGA), concanavalin A (Con A), *Ulex europaeus* agglutinin I (UEA-I), soybean agglutinin (SBA), and peanut agglutinin (PNA), were evaluated for the binding to different kinds of carbohydrate chips. Clearly, the results show that the predicted lectin selectively binds to the carbohydrates, which demonstrates the applicability of the approach. Furthermore, the kinetics of the interactions between Con A and mannose, WGA and *N*-Acetylglucosamine were studied, respectively. This study provides an efficient approach to preparing carbohydrate chips based on PDA for the lectin–carbohydrate interactions study.

## 1. Introduction

Carbohydrates located on the surfaces of cells play a crucial role in cell interactions, cell communication, cell proliferation, as well as cell death, where the interactions between carbohydrates and proteins are identified to be essential important in biological processes [1,2,3]. To date, several methods have been used to analyze the interactions between carbohydrates and proteins, such as enzyme-linked lectin assays (ELLAs), mass-spectrometric techniques, nuclear magnetic resonance, X-ray crystallography, microarray technologies, and biosensors [4,5,6,7,8,9,10,11,12]. Among them, biosensors are well known to its label-free and real-time detection, which have been wildly used to study biomolecular interactions. The quartz crystal microbalance (QCM) biosensor, which is based on the effect of piezoelectric, has turned out to be an efficient analytical tool for the assessment of carbohydrate–protein interactions with protein chips, carbohydrate chips, and cell chips [13,14,15,16,17,18,19]. Recently, as a useful analytical tool, QCM carbohydrate chip technology has attracted wide attentions for the carbohydrate–protein interaction study [20,21,22].

For the preparation of QCM carbohydrate chips, the target carbohydrate molecules must be immobilized on the surface of the chip [23,24]. Non-covalent immobilization refers to make the carbohydrate molecules conjugate to the basal surface by non-covalent function [14]. Pei et al. [25] immobilized carbohydrate molecules with non-covalent interaction by using the hydrophobic effect. However, non-covalent immobilization has limitations for the poor stability of this fixation. Covalent immobilization is the preparation of carbohydrate chips with covalent bonding between carbohydrate and substrate, which has more stability and wider application compared with non-covalent bonding. Using covalent immobilization, Seto et al. [26] prepared the carbohydrate chip which can be successfully used to recognition of lectin. Moreover, Oscar et al. [27,28] prepared a rapidly functionalized carbohydrate chip, which was the covalent immobilization of carbohydrate on the surface of polymer chips via click reaction and the PFPA azide photoligation. 

Polydopamine (PDA), an excellent surface modification material, has been widely used in chemistry, biology, medicine, as well as materials science [29,30,31]. In alkaline conditions, the catechol group in PDA can be oxidized to the phthalquinone, and the phthalquinone can react with the nucleophilic amino group or sulfhydryl group through Michael addition or Schiff base reaction [32,33]. Various functional groups, including catechol, phthalide, amino, and imino groups, can be used for secondary modification, laying a good foundation for the immobilization of biomolecules on PDA [32]. Molecules with sulfhydryl or amino groups will be quickly fixed when dissolved in an alkaline solution and incubated at room temperature on the surface of PDA. There are more advantages of this method such as mild reaction conditions, simple operation, wide application on solid base surfaces, compared with traditional method [31,34]. In our previous study, a protein chip on the surface of rapid functional chip using PDA was successfully prepared, which was used to analyze the kinetics of protein-protein binding process [35].

In this study, we have prepared carbohydrate chips based on PDA surface to evaluate carbohydrate–lectin interactions by QCM. Three monosaccharides including mannose (Man), galactose (Gal), and *N*-acetylglucosamine (GlcNAc) were selected for amination to yield aminated monosaccharide derivatives. Subsequently, theaminated monosaccharide derivatives were covalently immobilized on the surface of PDA coated chip via Schiff base reaction and/or Michael addition reactions, respectively, to give carbohydrate chips, which were used for the study of interaction and kinetics of carbohydrate and protein. This research provides a novel way to preparing carbohydrate chips based on PDA for the study of lectin–carbohydrate interactions.

## 2. Materials and Methods

### 2.1. Materials

*N*-Acetyl-d-glucosamine, trichloroacetonitrile, and p-Toluenesulfonic acid were purchased from Sun Chemical Technology Co. (Shanghai, China). d-mannose and 2-[2-(2-chloroethoxy) ethoxy] ethanol were purchased from Jiu Ding Chemistry Reagent Co. (Shanghai, China). d-Galactose and Pd-on-carbon were purchased from Adamas Reagent Co. (Shanghai, China) and Shanxi Rock New Materials Co. (Baoji, China) respectively. Boron trifluoride etherate, 1,8-diazabicyclo [5.4.0] undec-7-ene (DBU), sodium methanolate and amberlite IR-120 H^+^ resin were purchased from Aladdin Reagent Co. (Shanghai, China). Molecular sieves type 3Å was purchased from Sinopharm Chemical Reagent Co. (Shanghai, China). Tris(hydroxymethyl) amino-methane (Tris), fluorescein isothiocyanate-conjugated Con A (Con A-FITC), dopamine hydrochloride, bovine serum albumin (BSA), glycine, and sodium hydroxide were obtained from Sigma-Aldrich (St. Louis, MO, USA). WGA, UEA-I, PNA, and SBA were purchased from Vector Laboratories (Burlingame, CA, USA). Ethanolamine (1 M, pH 8.5) was provided by Attana (Stockholm, Sweden). Ultrapure water was used in this work. The running buffer of Attana Cell A200 QCM biosensor instrument (Attana AB, Stockholm, Sweden) was phosphate buffered saline. Other reagents with analytical grade were purchased from local suppliers, which were used without further purification.

**Figure 1:**
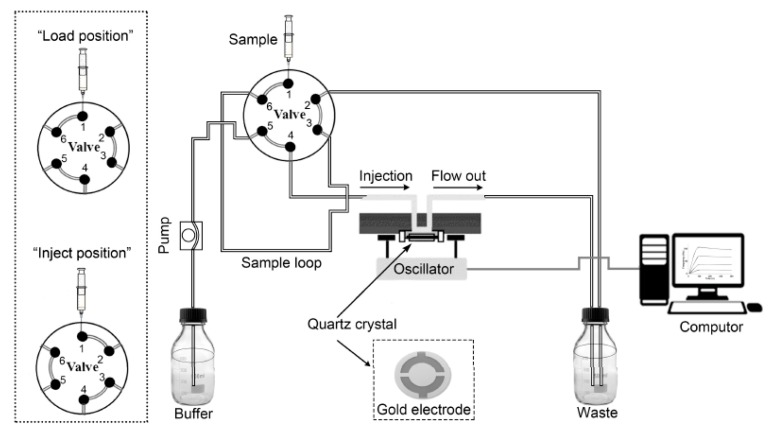
Continuous flow system of the Attana QCM biosensor [15].

Bruker 500 MHz Spectrometer (BrukerDaltonics Inc., Billerica, MA, USA) recorded ^1^H NMR spectra, using residual signals from D_2_O (^1^H: δ 4.79 ppm) or CDCl_3_ (^1^H: δ 7.26 ppm) as internal standards. Chemical composition of the sensor surface was determined by an X-ray photoelectron spectroscopy (XPS) instrument (Thermo Fisher Scientific Inc., Waltham, MA, USA). The Attana Cell A200 QCM (Attana AB, Stockholm, Sweden) under a continuous flow mode was used to carry out the interaction measurements (Figure 1) [15]. Attana gold sensor chips (Attana, Stockholm, Sweden), with 8 mm in diameter and a 4.5-mm diameter gold electrode on each side, were used for further modification to produce the carbohydrate sensor chips.

### 2.2. Gold Surface Functionalization by PDA Coating

The gold chip surface was cleaned for 30 min in a piranha solution of 30% H_2_O_2_ and H_2_SO_4_ (1:3 *v*/*v*) at 80 °C, and thoroughly rinsed with ultrapure water and dried under nitrogen stream. Then, 50 μL dopamine solution (2 mg/mL in 10 mM Tris buffer, pH 8.5) was added to the gold surface at room temperature for 30 min. After incubation in dopamine solution, the chip surface was operated by rinsing with ultrapure water and drying under nitrogen [35,36].

### 2.3. Synthesis of Amino-Carbohydrate

Compound **6**, **14**, and **20** were synthesized via previously reported procedures [37,38,39], as shown in Scheme S1–S3 in the Supporting Information, and their NMR data were displayed in Figures S1–S3, respectively.

### 2.4. Immobilization of Amino-Carbohydrate on the PDA-Coated Surface

50 μL carbohydrate solution (100 mg/mL in PBS, pH 7.4) was applied to the chip surface coated by PDA, then incubated for 4 h at room temperature. After incubation with the carbohydrate solution, the sensor chip surface was further rinsed with PBS in order to remove the carbohydrate which was un-immobilized on the surface.

### 2.5. XPS Measurement

In order to evaluate the chemical composition of the sensor chip surface, XPS spectra of these samples were obtained after drying the samples overnight.

### 2.6. Analysis of Lectin-Carbohyhdrate Interactions by QCM

The carbohydrate sensor chip was inserted to the Attana Cell A200 QCM biosensor, then stabilized under PBS running buffer (pH 7.4, 10 mM) in continuous flow (100 μL/min). When a stable baseline was achieved (frequency shift < 0.2 Hz/min), the flowrate was adjusted to 25 μL/min. To block the PDA areas which were not react with carbohydrate, BSA (50 μg/mL) was subsequently injected over the PDA-coated carbohydrate chip surface until the surface was saturated. After the injection of the BSA, the measurements of interactions can be recorded. The lectin diluted in running buffer at 50 μg/mL was injected to the chip surface. The association time is 84 s, and the dissociation time is 300 s. After each association and dissociation cycle, the chip surface regeneration was performed with injection of 10 mM Glycine (pH 1.5), and the bound analyte was removed. The recorded data was analyzed using the evaluation software of the Attana Cell A200. The kinetic evaluations were calculated by fitting the curves with a theoretical 1:1 binding model. The curve fitting data were used to interpret the kinetic constants, including the association rate constant (*k*_on_), the dissociation rate constant (*k*_off_), and the equilibrium dissociation constant (*K*_D_). ClampXP, developed by Tom Morton and David Myszka, is a global analysis of the association and dissociation phases of the interaction by fitting a theoretical 1:1 interaction model compensated for mass-transport limitation [17] and used for the biosensor kinetic data analysis.

### 2.7. Reuse of the Sensor Chip

The PDA coating was removed after the measurement with QCM according to Doriane Del Frari’s method [40]. Briefly, the sensor chip was immersed in NaClO solution (1 g/L) and then washed with ultrapure water. The chip can be reused for fabrication of PDA coated chips by following the protocol in Section 2.2.

## 3. Results and Discussion

**Figure 2:**
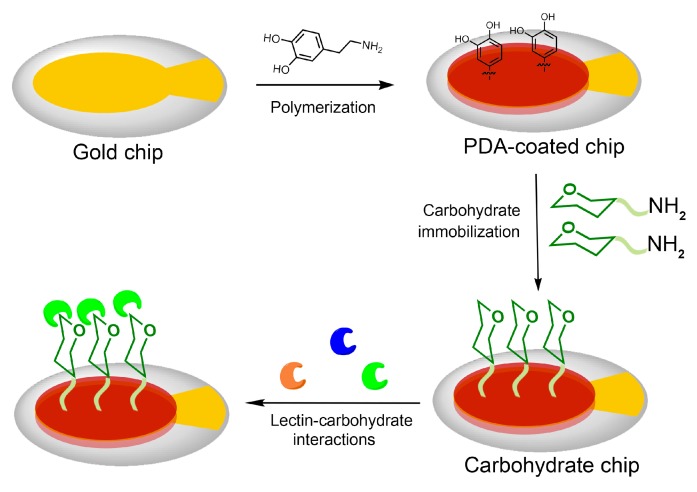
Schematic illustration of carbohydrate chip fabrication and interaction with lectins.

The fabrication of the carbohydrate chips and their interaction with lectins were illustrated in Figure 2. The carbohydrate chips were readily prepared using a two-step process. Firstly, a QCM gold sensor surface was coated with PDA in alkaline environment through the dopamine self-polymerization. Following PDA coating, the aminated carbohydrates were immobilized on the PDA coated chip surface via Schiff base and/or Michael addition reaction to yield the carbohydrate chips, respectively. The interactions between the lectins and the carbohydrates were evaluated by a QCM biosensor. In order to study the interaction of the immobilized carbohydrate on the sensor surface with its interacting lectin, the flow rate was set to 25 μL/min. The interacting lectin was diluted in running buffer and injected over the surface. The resonant frequency of the quartz crystal and the frequency shift (Δf) coupled to the association or dissociation were recorded with the Attester software in real time. The data were analyzed using Attester Evaluation software, where the biosensor kinetic data analysis was performed by using software ClampXP v3.50 [17]. 

### 3.1. XPS Analysis of the Sensor Chip Surface

**Figure 3:**
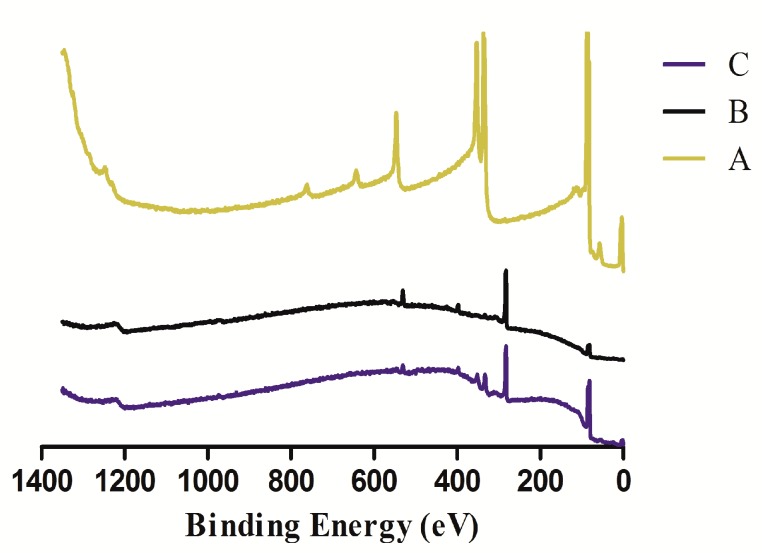
XPS spectra of different chip surfaces: unmodified gold chip surface (A), PDA-coated chip surface (B), and Compound **6**-immobilized chip surface (C).

XPS was used to analyze the chemical composition of these chip surfaces and the results were shown in Figure 3. From the analysis of XPS, compared to the Attana gold sensor chips, PDA-coated gold sensor chip was observed the disappearance of the photoelectron peaks of Au (56.7 eV for Au5p3, 84.2 eV for Au4f7, 87.9 eV for Au4f5, 111.8 eV for Au5s, 334.3 eV for Au4d5, 353.1 eV for Au4d3, 545.7 eV for Au4p3, 641.1 eV for Au4p1, and 762.1 eV for Au4s, Figure 3 A), and at the same time, the emergence of the peaks of carbon (C1s, 283.1 eV), oxygen (O1s, 531.5 eV), and nitrogen (N1s, 398.2 eV) (Figure 3 B). The XPS spectrum indicated evident signals for the atomic composition of PDA. The nitrogen-to-carbon molar ratio (N/C ratio) of the PDA-coated sensor chip surface is 0.120. The result is similar to the theoretical value of dopamine (N/C ratio = 0.125) and the same as the value reported [41]. These implied that the gold chip surface was coated completely with PDA.

Compared to the PDA-coated sensor chip, it was found that XPS spectrum signals of the sensor chip surface chemical composition (N1s, C1s, and O1s) changed after Compound **6** immobilized (Figure 3 C). The N/C ratio of the PDA-coated chip surface was 0.120. With the immobilization of Compound **6**, the ratio changed to 0.097, which is close to the theoretical value of Compound **6** (N/C = 0.083). These showed clearly that Compound **6** were immobilized on the PDA-coated chip surface.

### 3.2. Binding of FITC-Con A on the PDA Coated Carbohydrate Chip Surface

**Figure 4:**
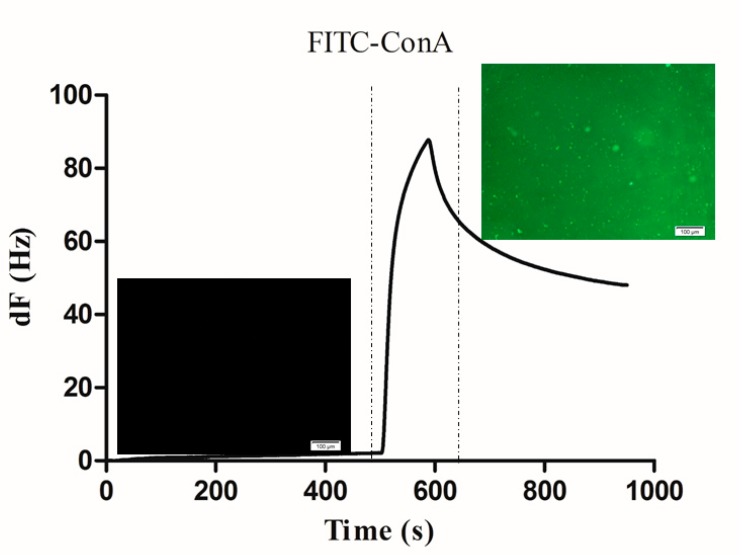
Binding of FITC-Con A to the carbohydrate chip. The scale bar is 100 µm.

When a stable baseline was achieved (frequency shift < 0.2 Hz/min), in order to block the PDA areas which was not modified with carbohydrate, bovine serum albumin (BSA) was repeatedly injected over the carbohydrate chip surface until the surface was saturated and no more binding could be detected. In order to prove the interaction between lectin and carbohydrate by fluorescence imaging, FITC-Con A with green fluorescence was used for the interaction study. After the injection of the BSA, FITC-Con A (50 μg/mL) was subsequently injected onto the sensor chip surface. As shown in Figure 4, the binding response of the injection of FITC-Con A was recorded, resulting in a frequency shift about 50 Hz. Before and after the injection of the FITC-Con A, a fluorescence microscopic evaluation was performed. Before injection, only a black field was observed, while green fluorescence could be observed after injection of FITC-Con A onto the carbohydrate chip surface. These different results confirmed that the frequency shift monitored by QCM specifically reflected the surface interactions between FITC-Con A and the carbohydrate which was immobilized on the PDA-coated surface.

### 3.3. QCM Measurements of Lectin–Carbohyhdrate Interactions

The method of fabrication of the carbohyhdrate sensor chip surface was evaluated using a QCM instrument. Three carbohydrates synthesized in the study were immobilized onto the sensor chip surface of PDA coating, and five different lectins were interacted with the three carbohydrates, respectively.

**Figure 5:**
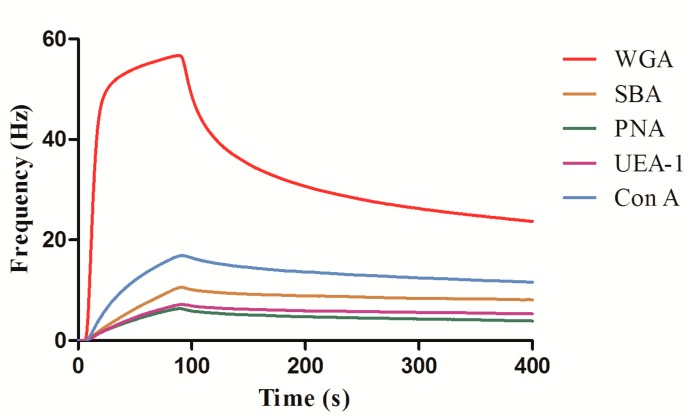
Five different lectin binding to GlcNAc-functionalized sensor chip surface. The data were collected after 400 s.

Firstly, the carbohydrate sensor chip was inserted to QCM, and then equilibrated under PBS running buffer at a flowrate of 25 μL/min. In order to minimize nonspecific binding, BSA solution was used to block the surfaces with several injections before the injection of lectins. When a stable baseline was achieved (frequency drift < 0.2 Hz/min), BSA (50 μg/mL) was injected to the carbohydrate chip surface. Then, an injection consisting of 50 μg/mL lectin (WGA, SBA, PNA, UEA-I, Con A) was performed in order to assess the interactions between the immobilized carbohydrate and the different lectins. Following each lectin injection, 10 mM glycine at pH 1.5 was injected to the surface to remove the interacting lectin. The processes of interaction and regeneration between lectin and carbohydrate were monitored using the Attester software. Figure 5 shows five different lectins (WGA, SBA, PNA, UEA-I, Con A) binding to GlcNAc-functionalized sensor chip surface. From the different lectin binding results, it can be noted that WGA proved to be the highest binding with more than 30 Hz based on binding specificity, small amounts of potential nonspecific lectin bindings were detected, hypothetically owing to nonspecific binding between unblocked PDA-coated carbohydrate sensor chip surface and lectins. 

**Figure 6:**
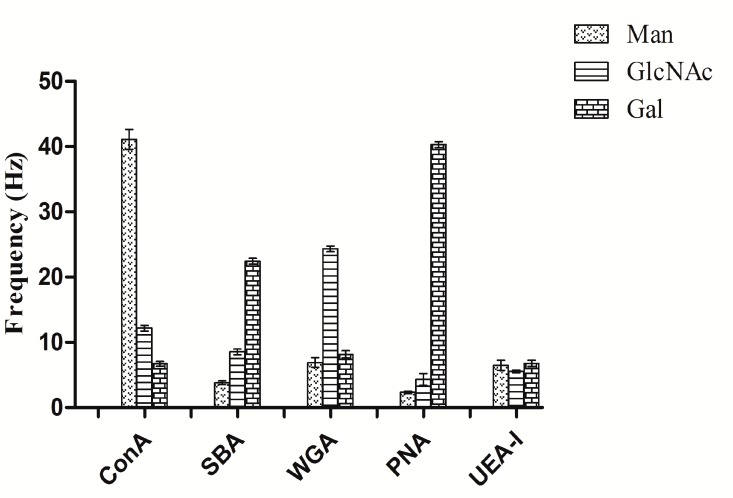
Binding between the five different lectins to the three carbohydrate sensor chip surfaces.

The five different lectins (WGA, SBA, PNA, UEA-I, Con A) binding to Man- and Gal-functionalized chip surfaces were also studied, respectively. The binding results between the different lectins and the three carbohydrate sensor chip surfaces were represented in Figure 6. From the binding analysis, Man-functionalized sensor chip surface displayed binding specificity to Con A, Gal-functionalized sensor chip surface displayed binding specificity to PNA. Comparison of the results in sensitivity among the three kinds of carbohydrate sensor chip surfaces shows that the surfaces had a specificity binding to different lectins, which is consistent with traditional ELLA test results [42]. Furthermore, the previous study of the interaction between the above-mentioned carbohydrates and lectins based on carbohydrate chips by other biosensors demonstrated the same specificity binding results [20,43,44]. 

### 3.4. Kinetic Studies

**Figure 7:**
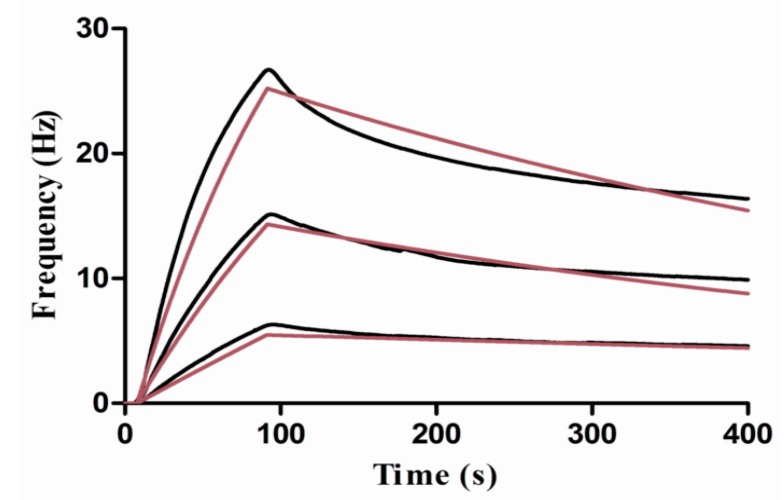
Kinetic evaluation of Con A and mannose interaction. The Con A at 20, 10, 5 μg/mL was injected over the sensor surface, respectively, and the measured responses were recorded as black lines, the theoretical 1:1 fits using the ClampXP software (Attana) were shown in red lines.

PDA-coated carbohydrate sensor surface was utilized to study the interaction kinetics between lectins and the immobilized carbohydrate. To study the interaction kinetic properties between Con A and mannose, a dilute solution of Con A in the running buffer was injected to the surface. The concentrations were 20, 10, and 5 μg/mL. The interaction between lectin and carbohydrate was monitored for 85 s association while for 300 s dissociation. After each cycle, 0.1 M glycine (pH 1.5) was injected for regeneration of the surface which enable remove the interacting lectin. Figure 7 shows the resulted binding responses. Using the ClampXP software, the global fit with the theoretical 1:1 interaction model was performed, giving the resultant association rate constant *k*_on_ = 3.51 × 10^4^ M^−1^·s^−1^, dissociation rate constant *k*_off_ = 1.60 × 10^−3^ s^−1^, and equilibrium dissociation constant *K*_D_ = 45.6 nM.

**Figure 8:**
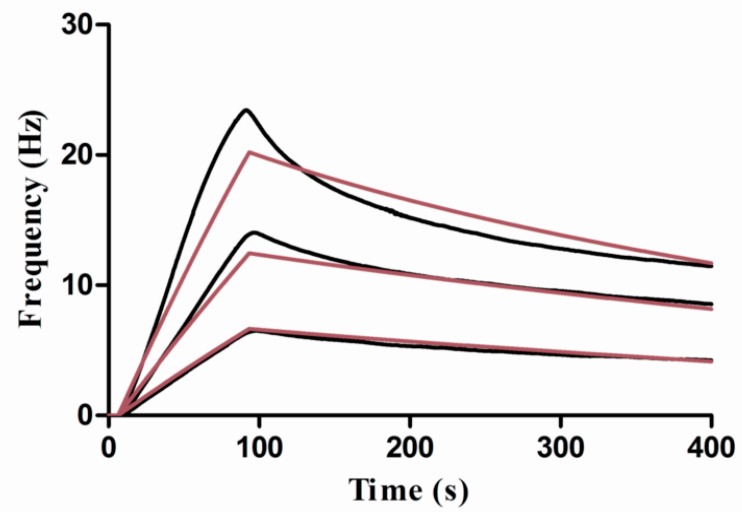
Kinetic evaluation of WGA and GlcNAc interaction. The WGA at 10, 5, 2 μg/mL was injected over the sensor surface, respectively, and the measured responses were recorded as black lines, the theoretical 1:1 fits using the ClampXP software (Attana) were shown in red lines.

In order to study the interaction affinity properties between WGA andGlcNAc, a dilute solution of WGA in the running buffer was injected to the surface. The concentrations were 10, 5, and 2 μg/mL. Figure 8 shows the measured responses, giving the resultant including the association rate constant *k*_on_ = 3.59 × 10^5^ M^−1^·s^−1^, dissociation rate constant *k*_off_ = 1.70 × 10^−2^ s^−1^, and equilibrium dissociation constant *K*_D_ = 47.2 nM.

## 4. Conclusions

In this study, a novel approach for fabricating carbohydrate chips based on PDA surface to determinate carbohydrate–lectin interactions by QCM was developed. Three kinds of carbohydrate chips were prepared by immobilizing the amino-monosaccharide derivatives on PDA-coated sensor surface. The surface via PDA coating was prepared through simple incubation by immersing the gold chip surface in the alkaline dopamine solution. Five kinds of plant lectins, including Con A, SBA, WGA, PNA, and UEA I, were evaluated for their binding to different kinds of carbohydrate chips, where the resulting frequency shifts show that the predicted lectin selectively bind to the respective carbohydrates. The further kinetic studies of the interactions between ConA and mannose, WGA and GlcNAc were performed, respectively. This work provides a good example for fabricating carbohydrate chips to evaluate carbohydrate–lectin interactions in real-time by QCM biosensor, which shows a potential application for studying biological processes.

## Supplementary Materials

The following are available online at http://www.mdpi.com/2073-4360/10/11/1275/s1. Scheme S1: Synthetic route of Compound **6**, Scheme S2: Synthetic route of Compound **14**, Scheme S3: Synthetic route of Compound **20**, Figure S1: ^1^H NMR spectrum (500 MHz, D_2_O) of **6**, Figure S2. ^1^H NMR spectrum (500 MHz, D_2_O) of **14**, Figure S3: ^1^H NMR spectrum (500 MHz, D_2_O) of **20**.

## References

[1] Park S., Lee M.R., Pyo S.J., Shin I. (2004). Carbohydrate chips for studying high-throughput carbohydrate-protein interactions. J. Am. Chem. Soc..

[2] Tyagi A., Wang X., Deng L., Ramström O., Yan M. (2010). Photogenerated carbohydrate microarrays to study carbohydrate-protein interactions using surface plasmon resonance imaging. Biosens. Bioelectron..

[3] Li X., Gao J., Liu D., Wang Z. (2011). Studying the interaction of carbohydrate-protein on the dendrimer-modified solid support by microarray-based plasmon resonance light scattering assay. Analyst.

[4] Gao J.Q., Liu D.J., Wang Z.X. (2008). Microarray-based study of carbohydrate-protein binding by gold nanoparticle probes. Anal. Chem..

[5] Gruber K., Horlacher T., Castelli R., Mader A., Seeberger P.H., Hermann B.A. (2011). Cantilever array sensors detect specific carbohydrate-protein interactions with picomolar sensitivity. ACS Nano.

[6] Harada S., Hiromori Y., Nakamura S., Kawahara K., Fukakusa S., Maruno T., Noda M., Uchiyama S., Fukui K., Nishikawa J. (2015). Structural basis for ppar gamma transactivation by endocrine-disrupting organotin compounds. Sci. Rep..

[7] Takahashi H., Nakanishi T., Kami K., Arata Y., Shimada I. (2000). A novel NMR method for determining the interfaces of large protein-protein complexes. Nat. Struct. Biol..

[8] Takeuchi K., Wagner G. (2006). NMR studies of protein interactions. Curr. Opin. Struct. Biol..

[9] Carlomagno T., Blommers M.J.J., Meiler J., Jahnke W., Schupp T., Petersen F., Schinzer D., Altmann K.H., Griesinger C. (2003). The high-resolution solution structure of epothilone a bound to tubulin: An understanding of the structure-activity relationships for a powerful class of antitumor agents. Angew. Chem. Int. Ed..

[10] Glish G.L., Vachet R.W. (2003). The basics of mass spectrometry in the twenty-first century. Nat. Rev. Drug. Discov..

[11] Li R.L., Lin C.W., Shao Y.L., Chang C.W., Yao F.K., Kowal M.D., Wang H.S., Yeung M.T., Huang S.C., Kaner R.B. (2016). Characterization of aniline tetramer by MALDI TOF mass spectrometry upon oxidative and reductive cycling. Polymers.

[12] Lee C.S., Muthusamy A., Abdul-Rahman P.S., Bhavanandan V.P., Hashim O.H. (2013). An improved lectin-based method for the detection of mucin-type *O*-glycans in biological samples. Analyst.

[13] Funari R., Della V.B., Altucci C., Offenhäusser A., Mayer D., Velotta R. (2016). Single molecule characterization of UV-activated antibodies on gold by atomic force microscopy. Langmuir.

[14] Pei Z., Aastrup T., Anderson H., Ramström O. (2005). Redox-responsive and calcium-dependent switching of glycosyldisulfide interactions with concanavalin A. Bioorg. Med. Chem. Lett..

[15] Song S., Lu Y., Li X., Cao S., Pei Y., Aastrup T., Pei Z. (2017). Optimization of 3D surfaces of dextran with different molecule weights for real-time detection of biomolecular interactions by a QCM biosensor. Polymers.

[16] Pei Z., Larsson R., Aastrup T., Anderson H., Lehn J.M., Ramström O. (2006). Quartz crystal microbalance bioaffinity sensor for rapid identification of glycosyldisulfide lectin inhibitors from a dynamic combinatorial library. Biosens. Bioelectron..

[17] Li X., Song S., Shuai Q., Pei Y., Aastrup T., Pei Y., Pei Z. (2015). Real-time and label-free analysis of binding thermodynamics of carbohydrate-protein interactions on unfixed cancer cell surfaces using a QCM biosensor. Sci. Rep..

[18] Li X., Pei Y., Zhang R., Shuai Q., Wang F., Aastrup T., Pei Z. (2013). A suspension-cell biosensor for real-time determination of binding kinetics of protein-carbohydrate interactions on cancer cell surfaces. Chem. Commun..

[19] Pei Z., Saint-Guirons J., Käck C., Ingemarsson B., Aastrup T. (2012). Real-time analysis of the carbohydrates on cell surfaces using a QCM biosensor: A lectin-based approach. Biosens. Bioelectron..

[20] Pei Y., Yu H., Pei Z., Theurer M., Ammer C., André S., Gabius H.J., Yan M., Ramström O. (2007). Photoderivatized polymer thin films at quartz crystal microbalance surfaces: Sensors for carbohydrate-protein interactions. Anal. Chem..

[21] Norberg O., Lee I.H., Aastrup T., Yan M., Ramström O. (2012). Photogenerated lectin sensors produced by thiol-ene/yne photo-click chemistry in aqueous solution. Biosens. Bioelectron..

[22] Lu Y., Song S., Hou C., Pang S., Li X., Wu X., Shao C., Pei Y., Pei Z. (2018). Facile fabrication of branched-chain carbohydrate chips for studying carbohydrate-protein interactions by QCM biosensor. Chin. Chem. Lett..

[23] Zhang Y., Luo S., Tang Y., Yu L., Hou K.Y., Cheng J.P., Zeng X., Wang P.G. (2006). Carbohydrate-protein interactions by “clicked” carbohydrate self-assembled monolayers. Anal. Chem..

[24] Uzawa H., Kamiya S., Minoura N., Dohi H., Nishida Y., Taguchi K., Yokoyama S., Mori H., Shimizu T., Kobayashi K. (2002). A quartz crystal microbalance method for rapid detection and differentiation of shiga toxins by applying a monoalkyl globobioside as the toxin ligand. Biomacromolecules.

[25] Pei Z., Anderson H., Aastrup T., Ramström O. (2005). Study of real-time lectin-carbohydrate interactions on the surface of a quartz crystal microbalance. Biosens. Bioelectron..

[26] Seto H., Ogata Y., Murakami T., Yu H., Miura Y. (2012). Selective protein separation using siliceous materials with a trimethoxysilane-containing glycopolymer. ACS Appl. Mater. Interfaces.

[27] Norberg O., Deng L., Aastrup T., Yan M., Ramström O. (2011). Photo-click immobilization on quartz crystal microbalance sensors for selective carbohydrate-protein interaction analyses. Anal. Chem..

[28] Norberg O., Deng L., Yan M., Ramström O. (2009). Photo-click immobilization of carbohydrates on polymeric surfaces—A quick method to functionalize surfaces for biomolecular recognition studies. Bioconjug. Chem..

[29] Liu Y., Ai K., Lu L. (2014). Polydopamine and its derivative materials: Synthesis and promising applications in energy, environmental, and biomedical fields. Chem. Rev..

[30] Wang K., Dong Y., Zhang W., Zhang S., Li J. (2017). Preparation of stable superhydrophobic coatings on wood substrate surfaces via mussel-Inspired polydopamine and electroless deposition methods. Polymers.

[31] Luo R., Tang L., Wang J., Zhao Y., Tu Q., Weng Y., Shen R., Huang N. (2013). Improved immobilization of biomolecules to quinone-rich polydopamine for efficient surface functionalization. Colloids Surf. B Biointerfaces.

[32] Lee H., Rho J., Messersmith P.B. (2009). Facile conjugation of biomolecules onto surfaces via mussel adhesive protein inspired coatings. Adv. Mater..

[33] Lei Z., Chen D., Hu W. (2016). Patterning of metal films on arbitrary substrates by using polydopamine as a UV-sensitive catalytic layer for electroless deposition. Langmuir.

[34] Frick C.P., Merkel D.R., Laursen C.M., Brinckmann S.A., Yakacki C.M. (2016). Copper-coated liquid-crystalline elastomer via bioinspired polydopamine adhesion and electroless deposition. Macromol. Rapid Commun..

[35] Wu C., Li X., Song S., Pei Y., Guo L., Pei Z., Wu C., Li X., Song S., Pei Y. (2017). QCM biosensor based on polydopamine surface for real-time analysis of the binding kinetics of protein-protein interactions. Polymers.

[36] Qiang Z., Akolawala S.A., Wang M. (2018). Simultaneous in-film polymer synthesis and self-assembly for hierarchical nanopatterns. ACS Macro Lett..

[37] Zhou J., Butchosa N., Jayawardena H.S., Zhou Q., Yan M., Ramström O. (2014). Glycan-functionalized fluorescent chitin nanocrystals for biorecognition applications. Bioconjug. Chem..

[38] Kato H., Uzawa H., Nagatsuka T., Kondo S., Sato K., Ohsawa I., Kanamorikataoka M., Takei Y., Ota S., Furuno M. (2011). Preparation and evaluation of lactose-modified monoliths for the adsorption and decontamination of plant toxins and lectins. Carbohydr. Res..

[39] Otman O., Boullanger P., Lafont D., Hamaide T. (2008). New amphiphilic glycopolymers based on a polycaprolactone-maleic anhydride copolymer backbone: characterization by ^15^N NMR and application to colloidal stabilization of nanoparticles. Macromol. Chem. Phys..

[40] Del Frari D., Bour J., Ball V., Toniazzo V., Ruch D. (2012). Degradation of polydopamine coatings by sodiumhypochlorite: A process depending on the substrate and the film synthesis method. Polym. Degrad. Stab..

[41] Lee H., Dellatore S.M., Miller W.M., Messersmith P.B. (2007). Mussel-inspired surface chemistry for multifunctional coatings. Science.

[42] Thompson R., Creavin A., O’Connell M., O’Connor B., Clarke P. (2011). Optimization of the enzyme-linked lectin assay for enhanced glycoprotein and glycoconjugate analysis. Anal. Biochem..

[43] Lienemann M., Paananen A., Boer H., de la Fuente J.M., García I., Penadés S., Koivula A. (2009). Characterization of the wheat germ agglutinin binding to self-assembled monolayers of neoglycoconjugates by AFM and SPR. Glycobiology.

[44] Vila-Perelló M., Gutiérrez Gallego R., Andreu D. (2005). A simple approach to well-defined sugar-coated surfaces for interaction studies. ChemBioChem.

