# Supplementary material for: Fabrication of Carbohydrate Chips Based on Polydopamine for Real-Time Determination of Carbohydrate–Lectin Interactions by QCM Biosensor
Source: Polymers (Basel). 2018 Nov 16;10(11):1275. doi: 10.3390/polym10111275 (PMC6401853; doi:10.3390/polym10111275)
Supplement: Supplementary file 1 [file polymers-10-01275-s001.pdf]

Supporting Information

Fabrication of carbohydrate chips based on polydopamine for real-time determination of carbohydrate-lectin interactions by QCM biosensor

Kun Shang[1,†], Siyu Song[1,†], Yaping Cheng[1], Lili Guo[1], Yuxin Pei[1,*], Xiaomeng Lv[1], Teodor Aastrup[2] and Zhichao Pei[1,*]

[1] Shaanxi Key Laboratory of Natural Products & Chemical Biology, College of Chemistry & Pharmacy, Northwest A&F University, Yangling 712100, China;

[2] Attana AB, SE-11419, Stockholm, Sweden

1. Materials and methods 2
2. Synthesis of 1-(2-(2-(2-Aminoethoxy)ethoxy)ethoxy-D-mannopyranoside (6) 2
3. Synthesis of 1-[2-{2-(2-Aminoethoxy)ethoxy}ethyl] 2-acetamido-2-deoxy-b-D-glucopyranoside (14) 7
4. Synthesis of 1-(2-(2-(2-Aminoethoxy)ethoxy)ethoxy-b-D-galactopyranoside (20) 12
5. Reference 15

1. Materials and methods

N-Acetyl-D-glucosamine, trichloroacetonitrile and p-Toluenesulfonic acid were purchased from Sun Chemical Technology Co. (Shanghai, China). D-mannose and 2-[2-(2-chloroethoxy) ethoxy] ethanol were purchased from Jiu Ding Chemistry Reagent Co. (Shanghai, China). D-Galactose and Pd-on-carbon were purchased from Adamas Reagent Co. (Shanghai, China) and Shanxi Rock New Materials Co. (Baoji, China) respectively. Boron trifluoride etherate, 1,8-diazabicyclo [5.4.0] undec-7-ene (DBU), sodium methanolate and amberlite IR-120 H+ resin were purchased from Aladdin Reagent Co. (Shanghai, China). Molecular sieves type 3Å was purchased from Sinopharm Chemical Reagent Co. (Shanghai, China). Other reagents were of analytical-reagent grade purchased from local suppliers, and used without further purification unless specified.

2. Synthesis of 1-(2-(2-(2-Aminoethoxy)ethoxy)ethoxy-D-mannopyranoside (6)

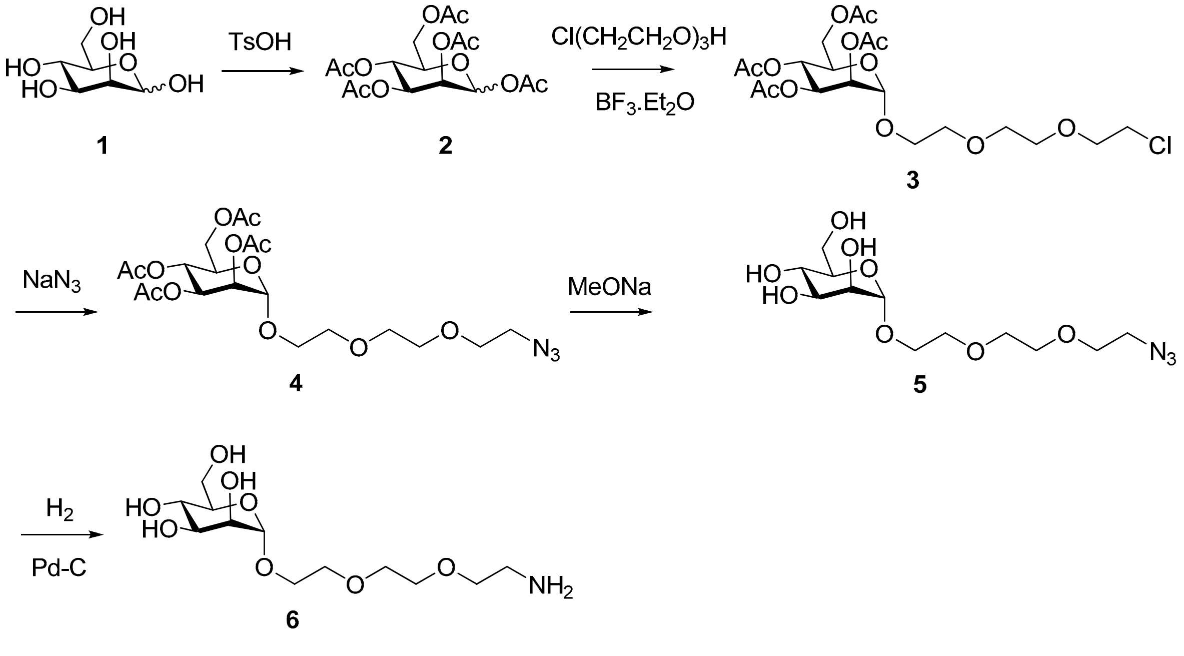

Scheme S1. Synthetic route of **6**.

*1,2,3,4,6- tetra-O-acetyl- D-mannopyranoside (**2**)*

To a 50 mL flask, D-mannose (Compound **1**) (2.0 g, 11.11 mmol) was added to a stirred liquid of acetic anhydride (10 mL), and the identical proportion of TsOH (1.913 g, 11.11 mmol) was added at 0 °C. The reaction mixture was stirred for 1 h, and then continued stirring at room temperature for about 12 hours. After completion of the reaction, the reaction mixture was monitored by TLC and poured into 30 mL icy water for quenching the

reaction. The aqueous phase was extracted with cold dichloromethane (3 × 40 mL). The combined organic phase was washed sequentially with cold saturated sodium bicarbonate solution (100 mL × 3), cold distilled water (100 mL × 3), and finally cold saturated brine (100 mL × 3). The organic phase was dried over Na_2SO_4, and then filtered. Dichloromethane was evaporated under diminished pressure to give a crude. Purification by silica-gel flash column chromatography using petroleum ether /ethyl acetate (5:2 v/v) as eluent gave **2** 3.825 g in 88.3% yield as a earthy yellow syrup, which was a mixture of the α and β anomers and used for the next step without further purification.

*2-(2-(2-chloroethoxy)ethoxy)ethoxy-2,3,4,6-tetra-O-acetyl-α-D-mannopyranoside (**3**)*

To a 50-mL flask with a solution of **2** (1.918 g, 4.91 mmol) in 12 mL anhydrous dichloromethane was added 2-[2-(2-chloroethoxy)ethoxy]ethanol (1.11 mL, 7.37 mmol) and freshly activated 3Å molecular sieves (1.0 g) at room temperature for 1 h under N_2 atmosphere. After the reaction mixture was cooled to 0°C, $BF_3{\cdot}EtO_2$ (1.8 mL, 14.75 mmol) was added. The mixture was stirred at 0° C for 48 h under N_2 atmosphere, and then quenched and neutralized with triethylamine, and filtered over a Celite bed. The filtrate was then diluted with dichloromethane (90 mL) and then washed successively with saturated sodium bicarbonate solution (3 × 100 mL), distilled water (100 mL × 3) and finally saturated brine (3 × 100 mL). After dryed over Na_2SO_4, the organic phase was filtered. Dichloromethane was removed in *vacuo* to give a crude, which was further purified by silica-gel flash column chromatography using petroleum ether/ethyl acetate (9:2 v/v) to give **3** as a yellow oil (0.838 g, 34.2%), which was directly used for the next step.[1]

*8-Azido-3,6-dioxaoctyl-2,3,4,6-tetra-O-acetyl-α-D-mannopyranoside (**4**)*

To a 25-mL flask witha suspension of sodium azide (0.0966 g, 1.485 mmol) in dry DMF (5 mL) was added Compound **3** (0.148 g, 0.3 mmol), and then stirred at 80 °C for 16 h under an argon atmosphere. After the reaction mixture was cooled to 0°C, the mixture was poured into 100 mL of icy water. The aqueous phase was extracted with dichloromethane (2 × 100 mL). The combined organic layers were washed sequentially with distilled water (200 mL × 3) and saturated brine (200 mL × 3). The organic phase was dried over Na_2SO_4, and then filtered. Dichloromethane was evaporated in *vacuo* to give a crude product. Purification by silica-gel flash column chromatography using petroleum ether/ethyl acetate (4:3 v/v) as eluent yielded **4** as a earthy yellow syrup (0.115 g, 76.3%), which was directly used for the next step.[2]

*8-Azido-3,6-dioxaoctyl-α-D-mannopyranoside (**5**)*

To a 25-mL flask with a solution of **5** (110 mg, 0.22 mmol) in methanol (5 mL) was added NaOMe (5.88 mg, 0.11 mmol), and then stirred overnight at room temperature. The reaction mixture was neutralized with Amberlite IR-120 H^+ resin and filtered. The residue was rinsed with methanol. The filtrate was collected and methanol was removed under diminished pressure to give a crude product. Purification by silica-gel flash column chromatography

using dichloromethane/methanol (15:1 v/v) as eluent gave **5** 71.47 mg in 97.4% yield as a clear syrup, which was directly used for the next step.[2]

*1-(2-(2-(2-Aminoethoxy)ethoxy)ethoxy-D-mannopyranoside (**6**)*

To a 10-mL flask with a solution of **5** (71.47 mg, 0.22 mmol) was added catalytic amount of Pd-on-carbon in methanol (3 mL). The flask was purged with N_2 and then filled with H_2 at room temperature. The mixture was stirred vigorously under atmospheric pressure for 8 h, and then filtered over a Celite bed. The solvent was removed under diminished pressure to give the product **6** (62.87 mg, 95.3%) as a white solid. ^{1}H NMR (500 MHz, D_2O) δ 4.85 (s, 1H), 3.93 (dd, J = 3.3, 1.6 Hz, 1H), 3.85 (d, J = 11.8 Hz, 2H), 3.80 – 3.65 (m, 10H), 3.64 – 3.59 (m, 2H), 3.17 (t, J = 5.6 Hz, 1H) ppm.[2]

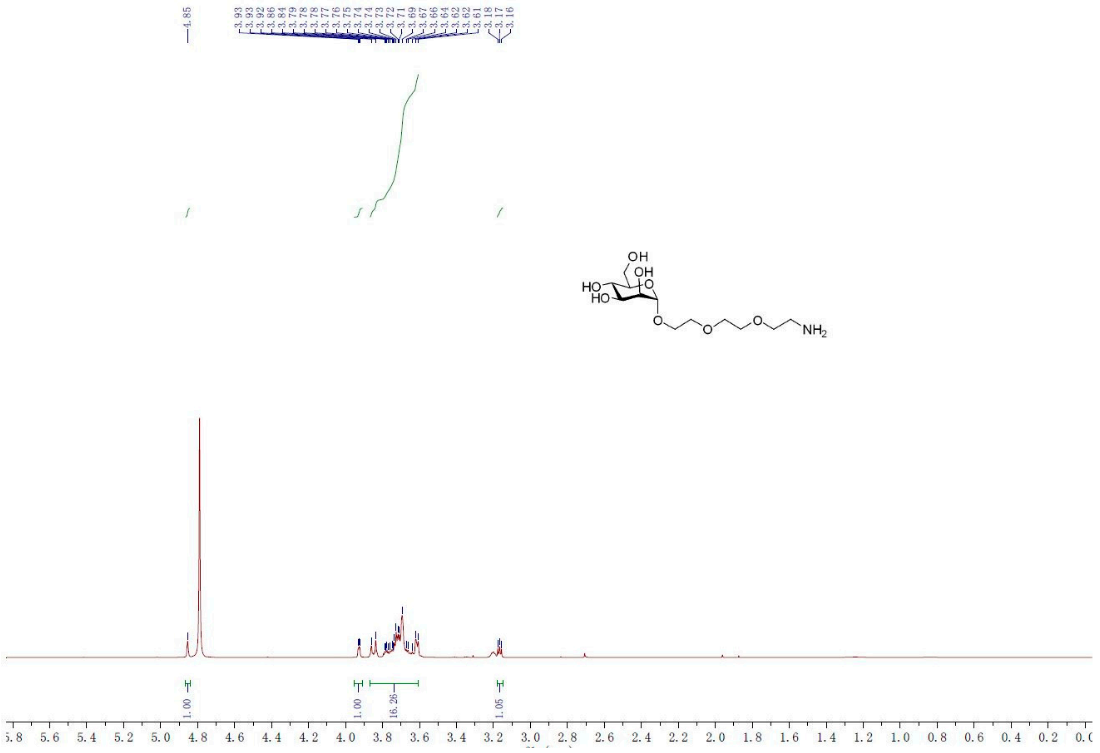

Figure S1. ^{1}H NMR spectrum (500 MHz, D_2O) of **6**.

3. *Synthesis of 1-[2-{2-(2-Aminoethoxy)ethoxy}ethyl] 2-acetamido-2-deoxy-β-D-glucopyranoside (14)*

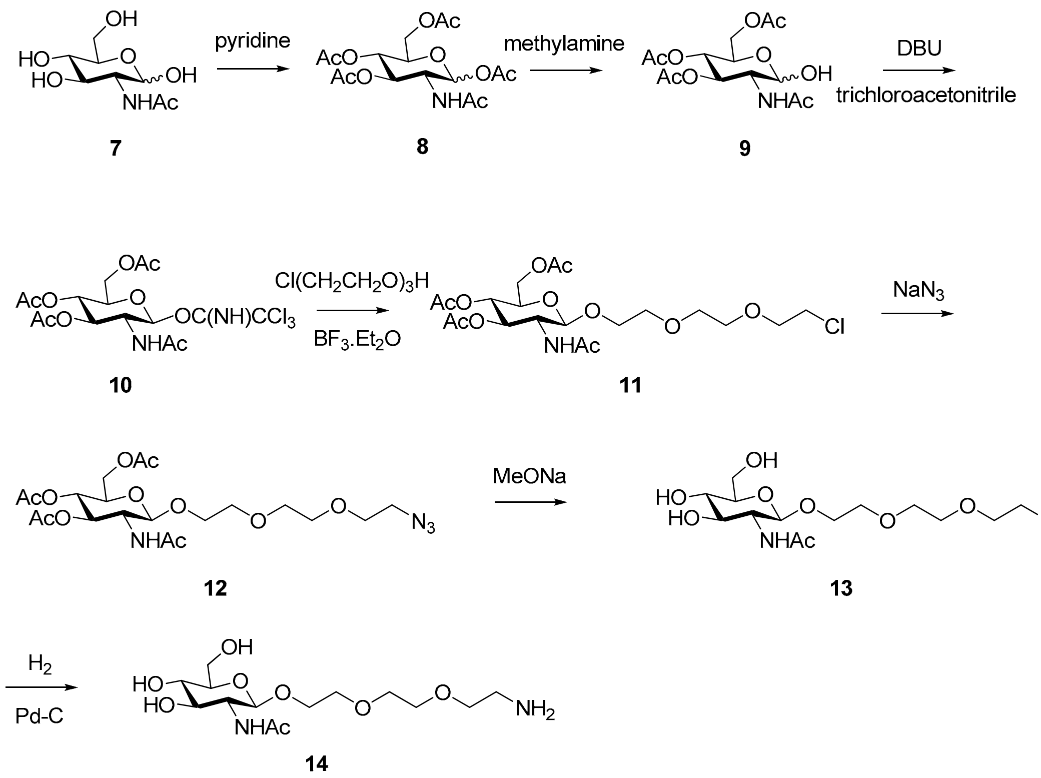

Scheme S2. Synthetic route of **14**.

*2-Acetamido-1,3,4,6-tetra-O-acetyl-2-deoxy-D-glucopyranose (**8**)*

To a 50-mL flask, Compound **7** N-Acetyl-D-glucosamine was added to a stirred mixture of 8 mL of anhydrous pyridine and 5 mL of acetic anhydride. The reaction mixture was stirred overnight at room temperature and then poured into 30 mL icy water for quenching the reaction. The aqueous phase was extracted with cold dichloromethane (3 × 40 mL), The combined organic layer was washed sequentially with cold saturated sodium bicarbonate solution (100 mL × 3), cold distilled water (100 mL × 3), and then with portions of a 10% solution of cupric sulfate until disappearance of the deep blue pyridine-copper complex, finally cold saturated brine (100 mL × 3). The organic phase was collected, dried over Na_2SO_4, and then filtered. Dichloromethane was removed under diminished pressure to give a crude product, which was used directly to the next step without purification.

*2-Acetamido-3,4,6-tri-O-acetyl-2-deoxy-D-glucopyranose (**9**)*

To a 100-mL flask with a solution of methylamine in methanol (2 M, 3.2 mL) was added a solution of **8** (1.2 g, 3.08 mmol) in THF (15 mL) at room temperature and the final mixture was stirred for 2 h. The reaction solvents were evaporated under diminished pressure to give a crude product. Purification by silica-gel flash column chromatography using petroleum ether/ethyl acetate (3:1 v/v) as eluentgave Compound **9** (0.818g, 76.4%) as a yellow syrup, which was a mixture of the α and β anomers and used for the next step without further purification.[3]

*2-Acetamido-3,4,6-tri-O-acetyl-2-deoxy-α-D-glucopyranosyl trichloroacetimidate (**10**)*

To a 50-mL flask, trichloroacetonitrile (0.95 mL, 11.96mmol) and DBU (0.07 mL, 0.47 mmol) were added to a stirred solution of **9** (0.82g, 2.36 mmol) in dry dichloromethane (13 mL). The final mixture was then stirred at room temperature till Compound **9** disappeared on TLC. Dichloromethane was removed under diminished pressure to give a crude product. Purification by silica-gel flash column chromatography using petroleum ether/ethyl acetate (7:1 v/v) as eluent gave **10** (0.72 g, 62.2%) as a light yellow solid, which was directly used for the next step.[3]

*2-[2-(2-chloroethoxy)ethoxy]ethyl-3,4,6-tri-O-acetyl-2-N-acetamido-2-deoxy-β-D-gluco pyranoside (**11**)*

To a 50-mL flask with a solution of **10** (0.71 g, 1.45 mmol) in 10 mL anhydrous dichloromethane were added 2-[2-(2-chloroethoxy)ethoxy]ethanol (0.3 mL, 2.17 mmol) and freshly activated 3Å molecular sieves (0.4 g) at room temperature for 1 h under N_2 atmosphere. After the reaction mixture was cooled to 0 °C, $BF_3{\cdot}EtO_2$ (0.5 mL, 4.34 mmol) was added. The solution mixture was stirred at 0 °C for 2-4 h under N_2 atmosphere, and then quenched and neutralized with triethylamine, and filtered over a Celite bed. The filtrate was then diluted with dichloromethane (90 mL) and then washed successively with saturated sodium bicarbonate solution (3 × 100 mL), distilled water (100 mL × 3) and finally saturated brine (3 × 100 mL). After dryed over Na_2SO_4, the organic phase was filtered. Dichloromethane was evaporated in vacuo to give a residue that was further purified by silica-gel flash column chromatography using petroleum ether/ethyl acetate (2:1 v/v) to give pure compound **11** as a yellow oil (0.331 g, 45.9%), which was directly used for the next step.[3]

*2-[2-(2-Azidoethoxy)ethoxy]ethyl-3,4,6-tri-O-acetyl-2-N-acetamido-2-deoxy-β-D-gluco pyranoside (**12**)*

The same experimental procedure as that used for the preparation of **4** from **3**, was applied to Compound **11** (0.149 g, 0.29 mmol) for the synthesis of **12**. After chromatographic purification (ethyl acetate/petroleum ether, 2:1 v/v), the expected compound **12** (0.135 g, 89.4% yield) was obtained as a yellow oil, which was directly used for the next step.[4]

*2-[2-(2-Azidoethoxy)ethoxy]ethyl -2-acetamido-2-deoxy-β-D-glucopyranoside (**13**)*

The same experimental procedure as that used for the preparation of **5** from **4**, was applied to Compound **12** (0.13 g, 0.26 mmol) for the synthesis of **13**. The expected compound **13** (0.0961 g, 98.5%) was obtained as a light yellow solid, which was directly used for the next step.[4]

1-[2-{2-(2-Aminoethoxy)ethoxy}ethyl] 2-acetamido-2- deoxy-β- D-glucopyranoside (**14**)

The same experimental procedure as that used for the preparation of **6** from **5**, was applied to Compound **13** (0.096 g, 0.25 mmol) for the synthesis of **14**. The expected compound **14** (0.0839 g, 93.7%) was obtained as a light yellow solid. ^{1}H NMR (500 MHz, D_2O) δ 4.55 (d, J = 8.5 Hz, 1H), 4.02 (ddd, J = 11.4, 5.8, 3.2 Hz, 1H), 3.95 – 3.91 (m, 1H), 3.74 (m, 12H), 3.57 – 3.53 (m, 1H), 3.45 (d, J = 6.5 Hz, 2H), 3.23 – 3.21 (m, 2H), 2.04 (s, 3H) ppm.[5]

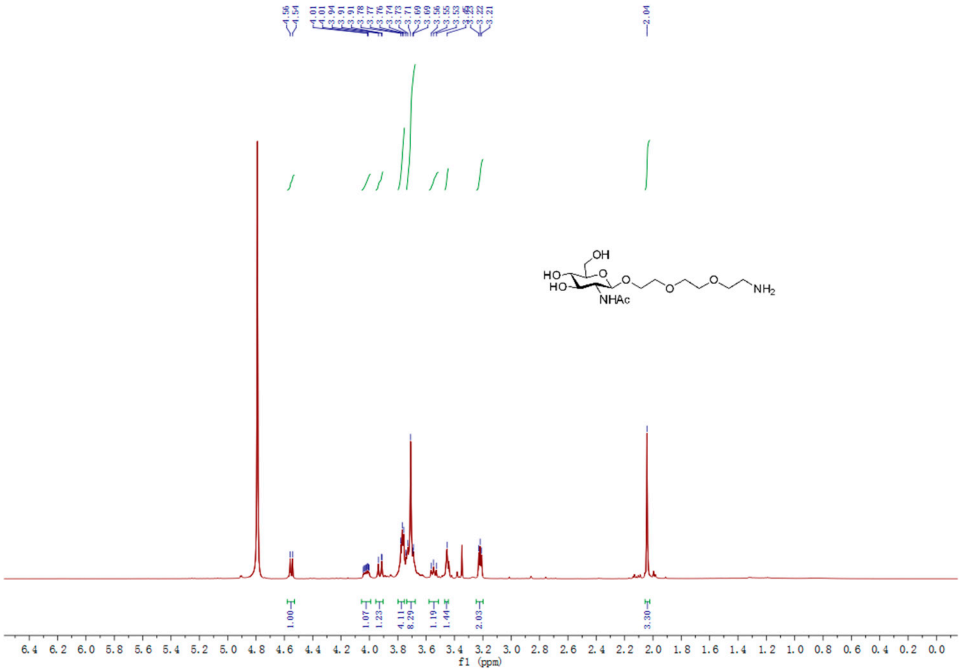

Figure S2. ^{1}H NMR spectrum (500 MHz, D_2O) of **14**.

4. Synthesis of 1-(2-(2-(2-Aminoethoxy)ethoxy)ethoxy-β-D-galactopyranoside (20)

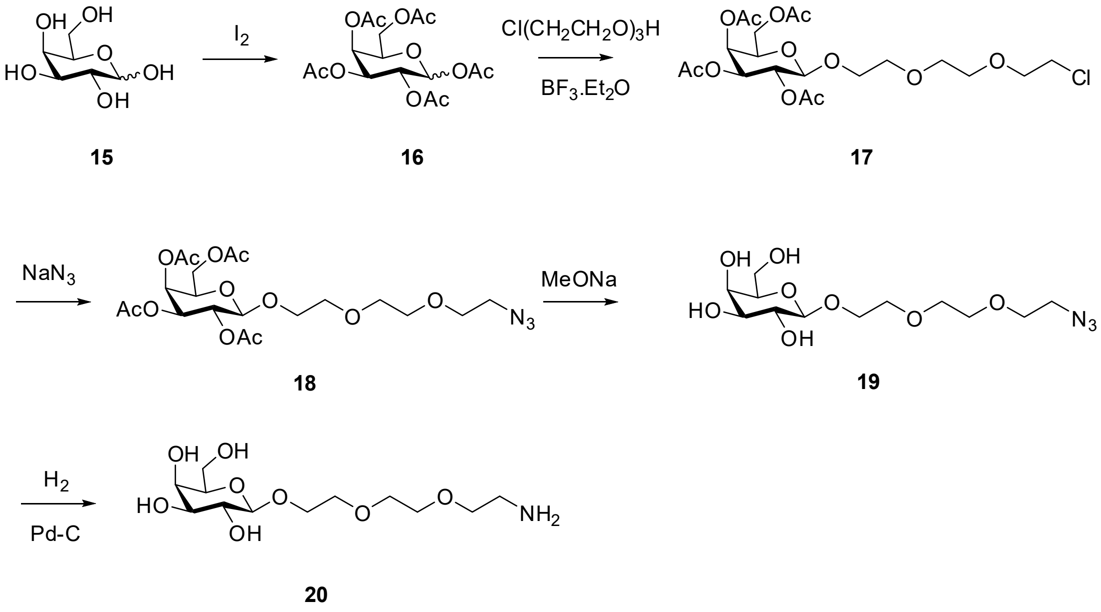

Scheme S3. Synthetic route of **20**.

*1,2,3,4,6-penta-O-acetate- D-galactopyranoside (**16**)*

To a 100-mL flask, D-Galactose (3.0 g, 16.66 mmol) was added to acetic anhydride (15 mL) under stirring, followed by the addition of iodine (0.166 g, 0.65 mmol) at 0 °C. The reaction mixture was stirred for 1 h, and followed by additional 8-h stirring at room temperature. At the end of the acetylation reaction, the reaction mixture was monitored by TLC and poured into 25 mL icy water for quenching the reaction. The aqueous phase was extracted with cold dichloromethane (3 × 40 mL), and then the combined organic phase was washed with saturated $Na_2S_2O_5$ solution (100 mL) to remove iodine. Afterwards, the organic layer was washed sequentially with cold saturated sodium bicarbonate solution (100 mL × 3), cold distilled water (100 mL × 3), and finally cold saturated brine (100 mL × 3). The organic phase was dried over anhydrous Na_2SO_4, and then filtered. Dichloromethane was removed under diminished pressure to give a crude product. Subsequently purification by silica-gel flash column chromatography using petroleum ether /ethyl acetate (2:1 v/v) as eluent gave **16** (6.49 g, 91.4%) as a white solid, which was a mixture of the α and β anomers and used for the next step without further purification.

*8-Chloro-3,6-dioxaoctyl 2,3,4,6-tetra-O-acetyl-β-D-galactopyranoside (**17**)*

The same experimental procedure as that used for the preparation of **3** from **2**, was applied to Compound **16** (4 g, 10.25 mmol) for the synthesis of **17**. After chromatographic purification (ethyl acetate/petroleum ether, 3:11 v/v), the expected compound **17** (1.01 g, 19.7%) was obtained as an earthy yellow oil, which was used directly to the next step.[5]

*8-Azido-3,6-dioxaoctyl 2,3,4,6-tetra-O-acetyl-β-D-galactopyranoside (**18**)*

The same experimental procedure as that used for the preparation of **4** from **3**, was applied to Compound **17** (1 g, 2.01 mmol) for the synthesis of **18**. After chromatographic purification (ethyl acetate/petroleum ether, 5:1 v/v), the expected compound **18** (0.593g, 58.5%) was obtained as a light yellow oil, which was used directly to the next step.[5]

*8-Azido-3,6-dioxaoctyl-β-D-galactopyranoside (**19**)*

The same experimental procedure as that used for the preparation of **5** from **4**, was applied to compound **18** (0.144 g, 0.29 mmol). The expected compound **19** (0.0876 g, 91.2%) was obtained as a light yellow oil, which was used directly to the next step.[5]

1-(2-(2-(2-Aminoethoxy)ethoxy)ethoxy-β-D-galactopyranoside *(**20**)*

The same experimental procedure as that used for the preparation of **6** from **5**, was applied to compound **19** (0.08 g, 0.24 mmol) for the synthesis of **20**. The expected compound **20**

(0.071g, 96.3%) was obtained as a clear oil. ^{1}H NMR (500 MHz, D_2O) δ 4.41 (d, J = 6.5 Hz, 1H), 4.01 - 4.12 (m, 1H), 3.90 (s, 1H), 3.85 – 3.49 (m, 14H), 3.33 (s, 1H), 2.90 (s, 1H) ppm.[6]

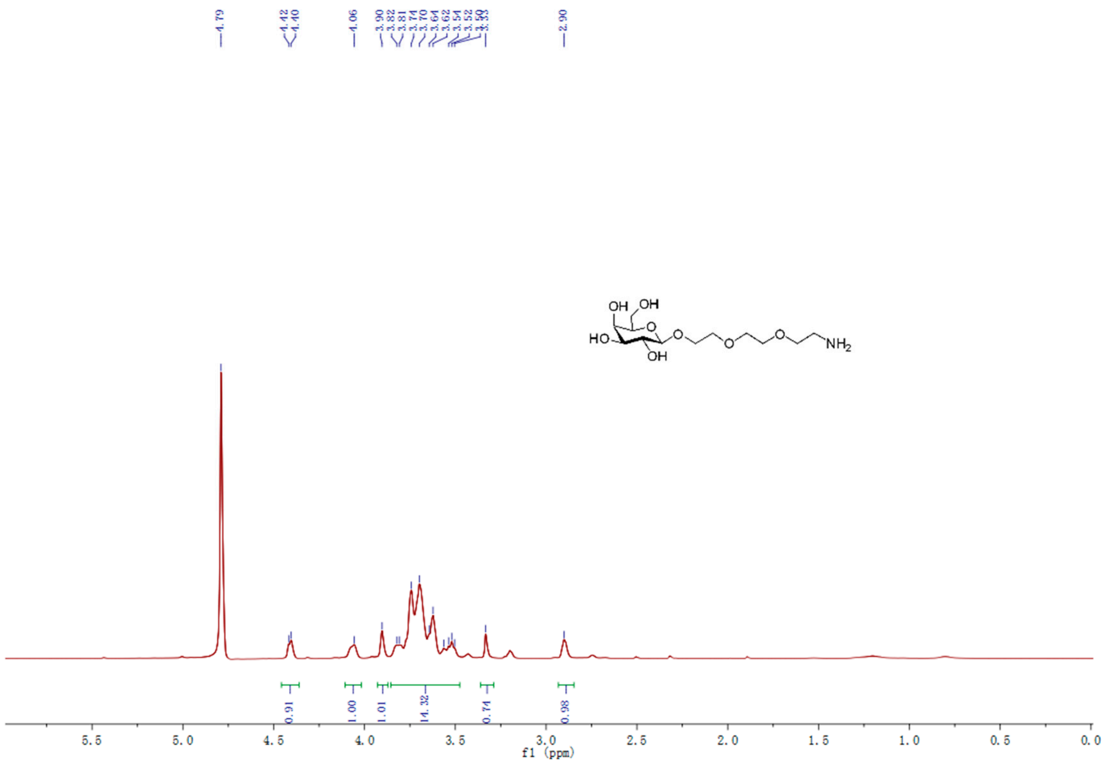

Figure S3. ^{1}H NMR spectrum (500 MHz, D_2O) of **20**

5. Reference

1. Dechtrirat D, Gajovic-Eichelmann N, Wojcik F, et al. Electrochemical displacement sensor based on ferrocene boronic acid tracer and immobilized glycan for saccharide binding proteins and E. coli. *Biosensors & Bioelectronics*, **2014**, *58*(8):1-8.

2. Zhou J, Butchosa N, Jayawardena H S, et al. Glycan-functionalized fluorescent chitin nanocrystals for biorecognition applications. *Bioconjugate Chemistry*, **2014**, *25*(4):640-643.

3. Gorityala B K, Lu Z, Leow M L, et al. Design of a "turn-off/turn-on" biosensor: understanding carbohydrate-lectin interactions for use in noncovalent drug delivery. *Journal of the American Chemical Society*, **2012**, *134*(37):15229-15232.

4. Amvam-Zollo P H, Sinaÿ P. Streptococcus pneumoniae type XIV polysaccharide: synthesis of a repeating branched tetrasaccharide with dioxa-type spacer-arms. *Carbohydrate Research*, **1986**, *150*(1):199-212.

5. Kato H, Uzawa H, Nagatsuka T, et al. Preparation and evaluation of lactose-modified

monoliths for the adsorption and decontamination of plant toxins and lectins. *Carbohydrate Research*, **2011**, *346*(13):1820-1826.

6. Otman Otman, Paul Boullanger, Dominique Lafont, et al. New amphiphilic glycopolymers based on a polycaprolactone-maleic anhydride copolymer backbone: characterization by ^{15}N NMR and application to colloidal stabilization of nanoparticles. *Macromolecular Chemistry and Physics*, **2010**, *209*(23):2410-2422.
